# Causal association between systemic lupus erythematosus and the risk of migraine: A Mendelian randomization study

**DOI:** 10.1002/brb3.3417

**Published:** 2024-02-12

**Authors:** Meixuan Ren, Hangtian Yu, Bing Xiao, Yan Zhao, Jiewei Yan, Jianghong Liu

**Affiliations:** ^1^ Department of Neurology, Xuanwu Hospital Capital Medical University, National Center for Neurological Disorders Beijing People's Republic of China; ^2^ Department of Cardiology The Second Hospital of Hebei Medical University Shijiazhuang Hebei People's Republic of China

**Keywords:** Mendelian randomization, migraine with aura, migraine without aura, risk, single nucleotide polymorphisms, systemic lupus erythematosus

## Abstract

**Background:**

Numerous studies have found that patients with systemic lupus erythematosus (SLE) often have comorbid headache, especially migraine. However, the causal relationship between genetically determined SLE and migraine risk remains unclear. Therefore, we conducted a Mendelian randomization (MR) study to explore this causal association.

**Methods:**

Genome‐wide association studies (GWAS) provided the instrumental variables. We selected summary data from GWAS of SLE as exposure (5201 SLE patients and 9066 controls). Both outcome GWAS data were from the Finnish Gene GWAS, including migraine with aura, migraine with aura and triptan purchases, and migraine without aura. The main MR approach was inverse‐variance weighted. Pleiotropy and heterogeneity were detected using the MR pleiotropy residual sum and outlier, MR‐Egger intercept test, leave‐one‐out analysis, and Cochran's *Q* test.

**Results:**

There was a significant association between genetically predicted SLE susceptibility and increased risk of migraine with aura [odds ratio (OR) = 1.05, 95% confidence interval (CI) = 1.02–1.08, *p* = .001]. The result was consistent when the outcome was migraine with aura and triptan purchases [OR = 1.05, 95% CI = 1.02–1.08, *p* = .001]. However, we found no association between SLE and migraine without aura. Our MR study showed no pleiotropy or heterogeneity.

**Conclusions:**

Our study indicates that genetic susceptibility to SLE increases the incidence of migraine with aura but not migraine without aura. It is necessary for the routine evaluation and early recognition of migraine in patients with SLE in clinical settings.

## Graphical abstract

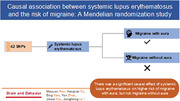

## 1 INTRODUCTION

Systemic lupus erythematosus (SLE) is a chronic autoimmune disease that can affect multiple organs and result in a wide range of clinical manifestations (Ainiala et al., 2001). A meta‐analysis reported a prevalence of 72.8 per 100,000 person‐years in the United States (Izmirly et al., 2021). Abnormal antibodies, immune complexes, complements, and cytokines all play roles in the clinical symptoms of SLE, ranging from mild joint pain and fatigue to potentially fatal organ damages (Kiriakidou & Ching, 2020). In 12%–95% of SLE patients, the central nervous system (CNS) is also affected with various clinical manifestations, including headache, especially migraine (ACR Ad Hoc Committee on Neuropsychiatric Lupus Nomenclature, 1999; Schwartz et al., 2019). Headache in SLE patients ranges from severe secondary headache (such as CNS infection, aseptic meningitis, and cerebral venous sinus thrombosis) to primary headache (such as migraine). Although not fatal, primary headache has a significant influence on individuals’ lives (Igor de Oliveira & Augusto, 2023).

Migraine is a primary headache disorder distinguished by recurring attacks of moderate to severe headache that may be accompanied by visual disturbances, vomiting, nausea, or sensitivity to certain stimuli, including photophobia and phonophobia. Based on the presence or absence of transient neurological disturbances preceding the onset of headache, migraine is defined as migraine with or without aura (Burch & Rayhill, 2018). Migraine is reported to affect millions of people globally and to be a serious health and financial burden (Steiner et al., 2020).

It has long been debated whether patients with SLE have a higher risk of migraine. In research, it has been estimated that 57.1% of SLE patients experience headache. Due to different definitions of SLE and migraine, the prevalence of migraine in SLE patients varies from 25% to 66.1% (Bettero et al., 2007; Lessa et al., 2006; Mitsikostas et al., 2004; Weder‐Cisneros et al., 2004). A 34‐month prospective follow‐up study of 27 SLE patients found that a history of migraine‐like headache was present in 67% of theSLE patients (Verro et al., 1998). A study of 80 SLE patients, 40 rheumatoid arthritis (RA) patients, and 40 controls showed that the prevalence of migraine was 42.5% in SLE patients and was significantly higher compared with RA patients and controls (Appenzeller & Costallat, 2004). A subsequent case–control study confirmed that the incidence of migraine was higher in patients with SLE than in healthy controls (Tjensvoll et al., 2011). In addition, a recent meta‐analysis showed that approximately one‐third of patients with SLE worldwide suffered from migraine and that the incidence of migraine was higher in patients with SLE than in healthy controls (Sarangi et al., 2023).

Further studies have shown the classification and clinical features of migraine in patients with SLE. A study of 81 patients with SLE in Mexico found a difference in the prevalence of migraine with aura (18%) and migraine without aura (7%) (Weder‐Cisneros et al., 2004). More severe headache and greater headache burden have been reported in patients with SLE than in controls without SLE (Tjensvoll et al., 2014). However, other studies found conflicting results, showing that the prevalence of migraine was similar between patients with SLE and controls. Migraine attacks were shorter and less severe in SLE patients (Katsiari et al., 2011).

There is a high familial incidence of migraine and SLE, reflecting an underlying genetic element (Appenzeller & Costallat, 2004). Therefore, it is reasonable to use genetics to improve our understanding of the association. Mendelian randomization (MR) is a promising approach for investigating a causal association. Single nucleotide polymorphisms (SNPs) of the traits of interest are used as instrumental variables (IVs) in MR analysis (Xu et al., 2022). Compared with conventional research, MR overcomes the bias of reverse causation and confounding factors (Plotnikov & Guggenheim, 2019). Genome‐wide association studies (GWAS) are publicly available and provide statistical summaries of IVs (Hartwig et al., 2016).

## 2 METHODS

### 2.1 MR design and data source

**FIGURE 1:**
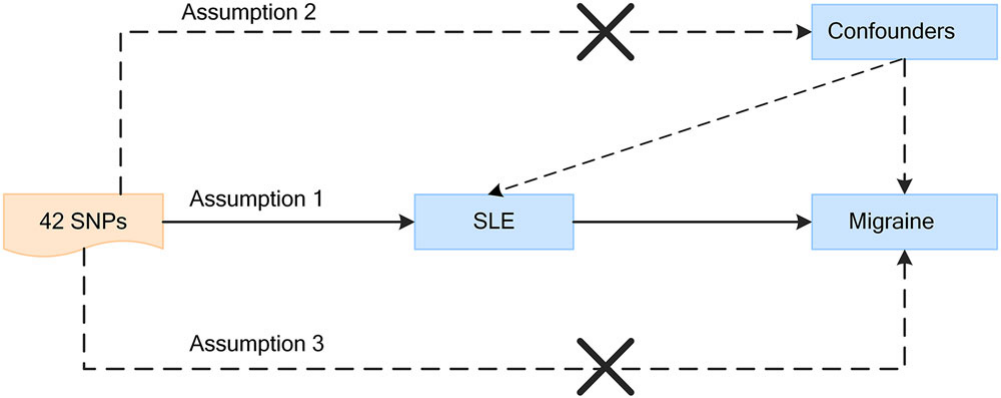
An overview of the design of our Mendelian randomization (MR) analysis. Dashed lines indicate causal associations that would go against the assumptions of MR. SLE, systemic lupus erythematosus; SNP, single nucleotide polymorphism.

A schematic of the MR design is shown in Figure 1. The genetic variants were used as IVs to explore the association between SLE and the risk of migraine. The data summarization of genetic variants was collected from GWAS and followed three assumptions (Emdin et al., 2017): (1) significantly associated with SLE; (2) not linked to confounders that affect the relationship between SLE and migraine; (3) influence the outcome through the exposure, not the mediators.

Details of the GWAS used in our analysis are shown in Table 1. All data were openly available and ethically approved in the original studies. The summary data for the phenotype of SLE were obtained from pooled GWAS (5201 SLE cases and 9066 controls) of European ancestry. Both outcome GWAS data were acquired from the Finnish Gene (FinnGen) GWAS, including migraine with aura (3541 cases and 176,107 controls), migraine with aura and triptan purchases (3541 cases and 164,089 controls), and migraine without aura (3215 cases and 176,107 controls).

**TABLE 1:** Details of the genome‐wide association studies (GWAS) used in the Mendelian randomization (MR) analysis.

Traits	Used as	Sample size (cases/controls)	Population	Data sources
SLE	Exposure	5201/9066	European	Bentham J et al.
Migraine with aura	Outcome	3541/176,107	European	FinnGen
Migraine with aura and triptan purchases	Outcome	3541/164,098	European	FinnGen
Migraine without aura	Outcome	3215/176,107	European	FinnGen

Abbreviations: FinnGen, Finnish Gene; SLE, systemic lupus erythematosus.

### 2.2 Selection of IVs

We extracted significant SNPs that met the genome‐wide significance threshold (*p* < 5 × 10^−8^) from the SLE GWAS. The SNPs in linkage disequilibrium (LD, *r*2 < .001 within a 10 Mb window) were excluded. We removed the IVs with an *F*‐statistic <10 to decrease the weak instrument bias (Burgess & Thompson, 2011). The formula for calculating the *F*‐statistic and *R* ^2^ is *F* = *R* ^2^/(1 − *R* ^2^) × (*N* − 2) and *R* ^2^ = 2 × beta^2^ × MAF × (1 − MAF). Palindromic variants were also excluded. Considering these strict criteria, we finally selected 45 SNPs for SLE. The details of the extracted SNPs are shown in Table S1.

### 2.3 Statistical analysis

**FIGURE 2:**
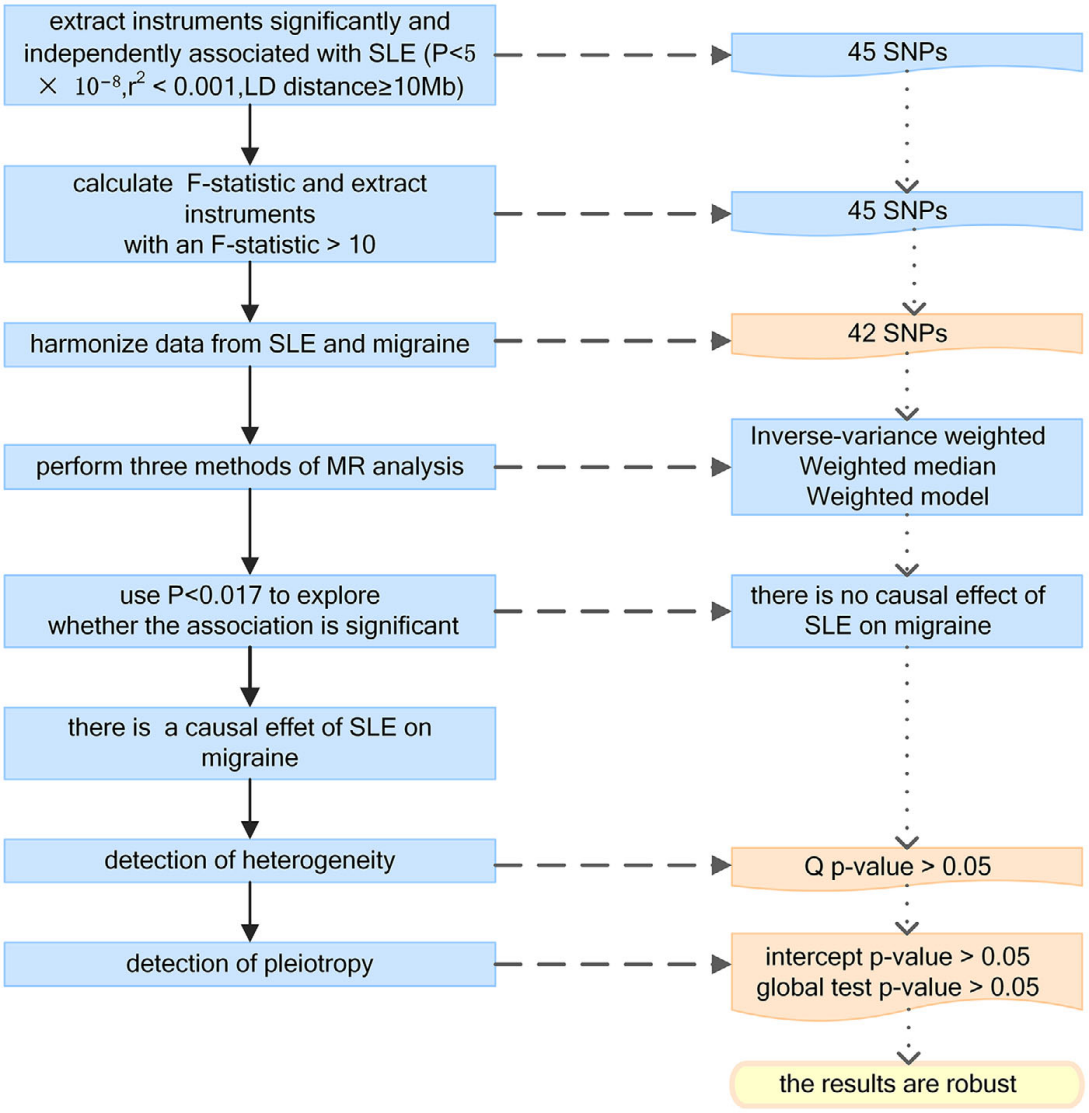
The flowchart of our Mendelian randomization (MR) study. LD, linkage disequilibrium; SLE, systemic lupus erythematosus; SNP, single nucleotide polymorphism.

To ensure the robustness and validity of the result, we performed multiple MR methods. Inverse‐variance weighted was the most important and powerful approach that integrated the effects of each SNP (Burgess et al., 2019). Under the condition that invalid variants were less than 50%, the weighted median method provided consistent results (Bowden et al., 2016). The weighted model method also provided an appropriate evaluation (Hartwig et al., 2017). An intercept *p*‐value in MR Egger <.05 or a global test *p*‐value in MR pleiotropy residual sum and outlier (MR‐PRESSO) <.05 was considered an indication of potential pleiotropy (Bowden et al., 2015; Verbanck et al., 2018). We also used Cochran's *Q* statistic to detect heterogeneity (Hemani et al., 2018). Finally, the leave‐one‐out chart was conducted for identifying SNPs that disproportionately influenced the result (Figures S1 and S2) (Hemani et al., 2018). The flowchart of our MR study is shown in Figure 2.

The R version 4.2.2 with the “Two‐Sample MR” and “MR‐PRESSO” packages was used to perform all statistical analyses (Hemani et al., 2018). We adjusted the significance *p*‐value for association to .017 using the Bonferroni method.

## 3 RESULTS

### 3.1 Causal effect of SLE on migraine with aura

**FIGURE 3:**
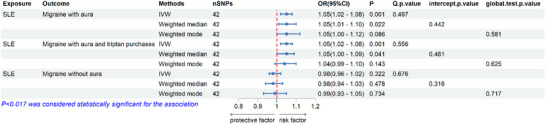
The results of the association between systemic lupus erythematosus (SLE) and migraine risk.

**FIGURE 4:**
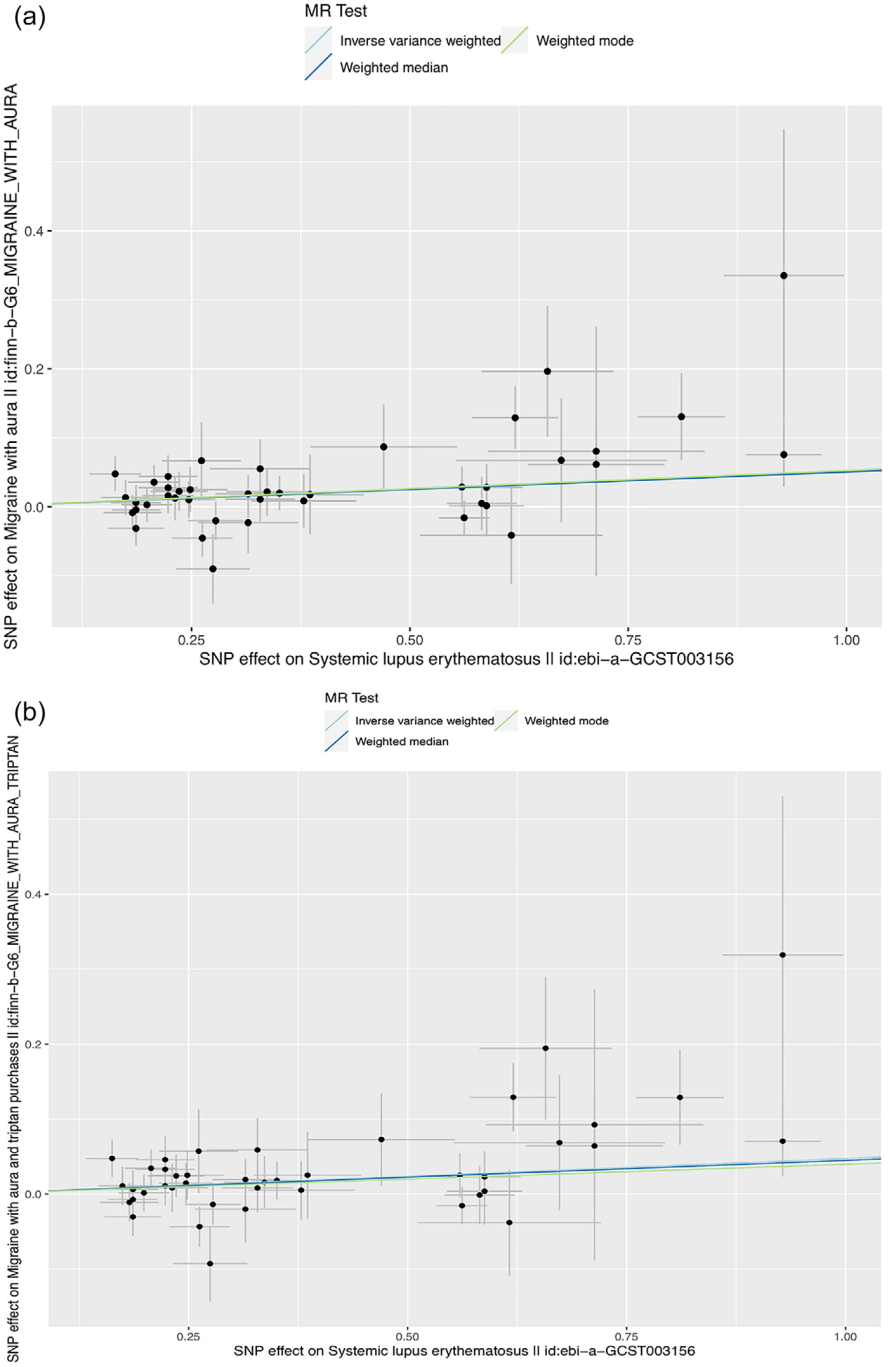
(a) Scatter plot of the causal effect of systemic lupus erythematosus (SLE) on the risk of migraine with aura; (b) scatter plot of the causal effect of SLE on the risk of migraine with aura and triptan purchases. SNP, single nucleotide polymorphism.

Details of the MR results are shown in Table 2 and Figure 3. There was a significant genetic causal effect of SLE on the increased risk of migraine with aura [odds ratio (OR) = 1.05, 95% confidence interval (CI) = 1.02–1.08, *p* = .001]. Cochran's *Q* statistic showed no heterogeneity (*Q p*‐value = .497). The leave‐one‐out analysis chart, the global test *p*‐value in MR‐PRESSO and the intercept *p*‐value in MR Egger revealed no significant pleiotropy (intercept *p*‐value = .442, global test *p*‐value = .581). The scatterplot of our MR analysis is presented in Figure 4.

**TABLE 2:** Details of the causal association between systemic lupus erythematosus (SLE) and migraine performed by Mendelian randomization (MR).

Exposure	Outcome	Methods	nSNPs	OR (95% CI)	*p*	*Q p*‐value	Intercept *p*‐value	Global test *p*‐value
SLE	Migraine with aura	IVW	42	1.05 (1.02–1.08)	**.001**	.497		
		Weighted median	42	1.05 (1.01–1.10)	.022		.442	
		Weighted mode	42	1.05 (1.00–1.12)	.086			.581
SLE	Migraine with aura and triptan purchases	IVW	42	1.05 (1.02–1.08)	**.001**	.556		
		Weighted median	42	1.05 (1.00–1.09)	.041		.461	
		Weighted mode	42	1.04 (.99–1.10)	.143			.625
SLE	Migraine without aura	IVW	42	.98 (.96–1.02)	.322	.676		
		Weighted median	42	.98 (.94–1.03)	.478		.316	
		Weighted mode	42	.99 (.93–1.05)	.734			.717

*Note*: Bold symbol indicates statistical significance (*p* < .017).

Abbreviations: CI, confidence interval; IVW, inverse variance‐weighted; MR‐PRESSO, Mendelian randomization pleiotropy residual sum and outlier; OR, odds ratio; SNP, single nucleotide polymorphism.

### 3.2 Causal effect of SLE on migraine with aura and triptan purchases

We found significant evidence that genetically predicted SLE susceptibility was linked to an increased risk of migraine with aura and triptan purchases [OR = 1.05, 95% CI = 1.02–1.08, *p* = .001]. The result was robust across several sensitivity analyses (*Q p*‐value = .556, intercept *p*‐value = .461, and global test *p*‐value = .625). Funnel plots of our MR analysis are shown in Figures S3 and S4.

### 3.3 Causal effect of SLE on migraine without aura

We found no evidence that the genetically determined SLE susceptibility was linked to migraine without aura [OR = .98, 95% CI = .96–1.02, *p* = .322]. The results were also supported by several sensitivity analyses (*Q p*‐value = .676, intercept *p*‐value = .316, and global test *p*‐value = .717).

## 4 DISCUSSION

Given the high prevalence of migraine in patients with SLE, we performed this MR study to investigate the causal association between SLE and migraine risk. The results revealed a significant association between genetically predicted SLE susceptibility and a higher risk of migraine with aura but not migraine without aura.

Previous observational studies have reported that different types of primary headache, especially migraine, are common neuropsychiatric manifestations in patients with SLE (Brey et al., 2002). Furthermore, several studies have also found that migraine with aura is more common than migraine without aura in patients with SLE, which is consistent with our findings (Mitsikostas et al., 2004; Weder‐Cisneros et al., 2004). At the level of clinical interpretation, MR is a method for causal inference and has several strengths compared with traditional observational studies. First, it has a larger sample size. Second, it can reveal a causal association, whereas observational studies cannot support a causal inference. Third, it can avoid the effect of confounders by using randomly assigned genetic variants as IVs (Plotnikov & Guggenheim, 2019; Xu et al., 2022). Several MR studies have attempted to investigate an association between SLE and neuropsychiatric disorders, including sleep traits, psychiatric disorders, and dementia. They found an association between SLE and a higher incidence of generalized anxiety disorder and schizophrenia, suggesting a genetic link between SLE and neuropsychiatric disorders (Jin et al., 2022; Sang et al., 2022; Xue et al., 2023). Our study is the first MR study to explore the causal effect of SLE on the risk of migraine subtypes separately and to elucidate the genetic and functional basis of this association.

The differential impact of genetic susceptibility to SLE on different subtypes of migraine deserves careful consideration. Possible explanations include shared genetic pathways that affect specific biological mechanisms associated with both SLE and migraine with aura, such as immunological, vascular, neural, or brain structural factors. Patients with SLE may have antiphospholipid antibodies (aPLs), including anti‐β2‐glycoprotein I (β2GPI) antibody, anticardiolipin (aCL) antibody, and lupus anticoagulant (Petri et al., 2012). Related studies have identified several mechanisms underlying aPLs‐mediated migraine. First, aPLs might contribute to the pathogenesis of migraine by regulating the number or proliferation of T cell subsets, such as T‐cytotoxic, T‐helper, and T‐regulatory cells (Islam et al., 2017). Second, it has been found that SLE patients with positive aCL and β2GPI have significantly elevated level of tumor necrosis factor‐α (TNF‐α) (Swadzba et al., 2011). Elevated plasma level of TNF‐α has also been seen in migraineurs and may contribute to the pathophysiology of migraine through several mechanisms, including increasing inflammatory reactions, sensitizing trigeminal ganglia, and activating the transcription of calcitonin gene‐related peptide (CGRP) (Durham, 2006; Fidan et al., 2006; Franceschini et al., 2013; Yücel et al., 2016). TNF‐α, trigeminal ganglia, and CGRP have been reported to be involved in the mechanism of migraine with aura (Shibata et al., 2023; Yamanaka et al., 2023). Therefore, it is possible that aCL and β2GPI contribute to migraine with aura via TNF‐α‐mediated pathways.

Previous studies have also linked migraine with aura to vascular disorders (Dalkara et al., 2010). It has been reported that the occurrence of migraine in SLE patients is associated with Raynaud's syndrome, implying the underlying vascular physiopathological mechanism (Dalkara et al., 2010). In a β2GPI‐dependent manner, aPLs bound to the surface of endothelial cells, activated them, and subsequently modulated several substances involved in the pathogenic mechanism of migraine, such as serotonin and endothelin‐1 (del Papa et al., 1997). It has been suggested that endothelin‐1 is involved in the development of vasoconstriction and leads to the aura of migraine (Hamed et al., 2010; Shima et al., 2021).

Migraine is also thought to be a neurological disorder characterized by abnormal neuronal electrical activity and the aura is caused by cortical spreading depression (CSD) (Lai & Dilli, 2020). CSD is defined as a period of electrical suppression of cortical neurons originating from the posterior cortex, slowly spreading to the nearby cortex and being accompanied by spreading oligemia (Dreier, 2011). Microglia have been found to be activated in patients with SLE and associated with CSD (Wang et al., 2022). On the one hand, a certain microglia‐regulated neuronal calcium response was required for the occurrence of CSD. On the other hand, microglia increased susceptibility to CSD (Shibata & Suzuki, 2017).

Neuroimaging studies have demonstrated the disruption of blood–brain barrier (BBB) in patients of migraine with aura (Cha et al., 2007). The aPLs could disrupt the BBB either directly by binding to neurons, astrocytes, and glial cells, or indirectly by activating endothelial cells and causing subsequent inflammation (Gris et al., 2015; Katzav et al., 2014). Besides, it was reported that SLE patients with migraine had a diffuse reduction in gray matter (GM) compared with SLE patients without migraine and healthy controls, indicating that unexplored pathways caused GM loss and increased migraine susceptibility in patients with SLE (Tjensvoll et al., 2016). Migraineurs with aura had a reduction in GM of different regions, which might be associated with different symptoms of aura (Petrusic et al., 2018). In eighteen SLE patients, brain perfusion single photon emission computed tomography found focal hypoperfusion or interictal hypofunction of the anterior cingulate cortex in some patients (Nobili et al., 2006). To our knowledge, the anterior cingulate cortex is a critical region in the network for the cortical elaboration of pain, suggesting that brain impairment may be another potential mechanism explaining the risk of migraine in patients with SLE (Matharu et al., 2004; Mechtler, 2004). One study found significantly decreased GM values in the bilateral dorsal part of the anterior cingulate cortex in migraineurs with aura compared with healthy controls (Hougaard et al., 2016).

Overall, genetic factors may contribute to individual differences in immunological, vascular, neurological, and brain structural characteristics of SLE patients, thereby increasing susceptibility to migraine with aura. These findings support the necessity of routine evaluation and early recognition of migraine in patients with SLE in clinical practice. This may help to avoid unnecessary invasive examinations and aggressive treatment and allow for appropriate therapies, ultimately improving patients’ quality of life (Igor de Oliveira & Augusto, 2023). However, due to the lack of support from clinical and basic research, these findings should be interpreted with caution. Further clinical and laboratory research is needed to explore the underlying mechanisms. Our MR study has several limitations. First, the findings may not apply to other populations because the GWAS data used in our study are based on European ancestry. Second, although we performed multiple sensitivity analyses, it is still possible that pleiotropy exists in our study. Third, both SLE and migraine may be influenced by hormones and are more common in women (Appenzeller & Costallat, 2004). However, the original study is not female‐specific. Fourth, previous studies have shown that active migraine and history of migraine have different clinical implications (Appenzeller & Costallat, 2004). However, the subgroup analysis was not performed because the data were not available. In addition, a headache diary should be used in future studies to accurately report headache features and reach a prospective conclusion.

## 5 CONCLUSIONS

To summarize, our study showed that genetically determined SLE increased the incidence of migraine with aura but not migraine without aura. Future research should investigate the underlying mechanism associating SLE and migraine with aura, which may lead to innovative therapeutic options targeting similar pathways.

## AUTHOR CONTRIBUTIONS

**Meixuan Ren**: Writing—original draft; conceptualization; methodology. **Hangtian Yu**: Software; formal analysis; data curation; methodology; visualization. **Bing Xiao**: Validation; supervision. **Yan Zhao**: Software; supervision. **Jiewei Yan**: Writing—original draft. **Jianghong Liu**: Writing—review and editing; funding acquisition; project administration.

## CONFLICT OF INTEREST STATEMENT

There are no conflicts of interest among all authors.

### PEER REVIEW

The peer review history for this article is available at https://publons.com/publon/10.1002/brb3.3417.

## Supporting information

Table S1 Characteristics of instrumental variables for Systemic Lupus Erythematosus.
Figure S1 MR leave‐one‐out sensitivity analysis for SLE on the risk of migraine with aura. MR, Mendelian randomization; SLE, systemic lupus erythematosus.
Figure S2 MR leave‐one‐out sensitivity analysis for SLE on the risk of migraine with aura and triptan purchases. MR, Mendelian randomization; SLE, systemic lupus erythematosus.
Figure S3 Funnel plot of causal effect of SLE on the risk of migraine with aura. IV, instrumental variable; MR, Mendelian randomization; SLE, systemic lupus erythematosus.
Figure S4 Funnel plot of causal effect of SLE on the risk of migraine with aura and triptan purchases. IV, instrumental variable; MR, Mendelian randomization; SLE, systemic lupus erythematosus.

## Data Availability

The summary‐level data for the exposures and outcomes were obtained from the Ieu Open Gwas Project at https://gwas.mrcieu.ac.uk/. All data for the exposures and outcomes are openly available and ethically approved in the original studies from their respective institutional review boards.
