# Supplementary material for: Causal association between systemic lupus erythematosus and the risk of migraine: A Mendelian randomization study
Source: Brain Behav. 2024 Feb 12;14(2):e3417. doi: 10.1002/brb3.3417 (PMC10861356; doi:10.1002/brb3.3417)
Supplement: Supplementary file 1 — Table S1 Characteristics of instrumental variables for Systemic Lupus Erythematosus. Figure S1 MR leave‐one‐out sensitivity analysis for SLE on the risk of migraine with aura. MR, Mendelian randomization; SLE, systemic lupus erythematosus. Figure S2 MR leave‐one‐out sensitivity analysis for SLE on the risk of migraine with aura and triptan purchases. MR, Mendelian randomization; SLE, systemic lupus erythematosus. Figure S3 Funnel plot of causal effect of SLE on the risk of migraine with aura. IV, instrumental variable; MR, Mendelian randomization; SLE, systemic lupus erythematosus. Figure S4 Funnel plot of causal effect of SLE on the risk of migraine with aura and triptan purchases. IV, instrumental variable; MR, Mendelian randomization; SLE, systemic lupus erythematosus. [file BRB3-14-e3417-s001.docx]

**Additional Information**

1. Characteristics of instrumental variables for Systemic Lupus Erythematosus

| **SNP** | **EA** | **OA** | **Gene** | **SE** | **Beta** | **EAF** | **P value** | **R^2^** | **F - statistic** |
| --- | --- | --- | --- | --- | --- | --- | --- | --- | --- |
| rs6679677 | A | C | PHTF1 | 0.0465 | 0.3365 | 0.0915 | 4.55E-13 | 0.0188 | 273.6516 |
| rs4661543 | G | T | KAZN | 0.0424 | 0.2744 | 0.8728 | 9.40E-11 | 0.0167 | 242.6125 |
| rs10912578 | G | A | snoU13 | 0.031 | -0.2469 | 0.3012 | 1.65E-15 | 0.0257 | 375.5757 |
| rs17849501 | T | C | NCF2 | 0.0499 | 0.8109 | 0.9404 | 1.81E-59 | 0.0737 | 1135.2271 |
| rs6671847 | A | G | FCGR2A | 0.029 | 0.1989 | 0.4871 | 6.64E-12 | 0.0198 | 287.5244 |
| rs4916215 | T | C | RP11-296O14.1 | 0.034 | 0.2231 | 0.2545 | 5.07E-11 | 0.0189 | 274.721 |
| rs12094036 | C | T | NCF2 | 0.0579 | -0.3285 | 0.0815 | 1.37E-08 | 0.0162 | 234.2577 |
| rs13019891 | T | G | IL1F10 | 0.029 | -0.5621 | 0.5487 | 1.65E-83 | 0.1565 | 2646.4787 |
| rs2573219 | C | A | ECEL1P1 | 0.0429 | 0.5878 | 0.0865 | 1.13E-42 | 0.0546 | 823.8552 |
| rs10200680 | T | C | KCNE4 | 0.0425 | -0.2485 | 0.8559 | 4.96E-09 | 0.0152 | 220.5817 |
| rs268124 | T | C | SPRED2 | 0.0324 | 0.1863 | 0.2744 | 8.60E-09 | 0.0138 | 199.9838 |
| rs2459611 | T | C | STAT4 | 0.0452 | 0.2614 | 0.1252 | 7.62E-09 | 0.015 | 216.6993 |
| rs4274624 | T | C | STAT4 | 0.0327 | -0.559`6 | 0.2316 | 9.73E-66 | 0.1115 | 1789.5057 |
| rs10048743 | T | G | IKZF2 | 0.0412 | -0.2311 | 0.1412 | 2.04E-08 | 0.013 | 187.213 |
| rs34703115 | C | T | SLC8A1-AS1 | 0.1048 | -0.6162 | 0.0328 | 4.08E-09 | 0.0241 | 352.1324 |
| rs1464446 | T | G | SIAH2 | 0.0401 | -0.3285 | 0.1789 | 2.79E-16 | 0.0317 | 467.0695 |
| rs9852014 | G | A | MRPL3 | 0.0493 | 0.6206 | 0.9254 | 2.26E-36 | 0.0532 | 801.1074 |
| rs13136219 | T | C | BANK1 | 0.0278 | -0.1744 | 0.6203 | 3.50E-10 | 0.0143 | 207.2368 |
| rs1078324 | A | C | PPARGC1B | 0.0782 | -0.7134 | 0.0497 | 7.11E-20 | 0.0481 | 720.3078 |
| rs4388254 | T | C | CTB-1I21.1 | 0.0604 | 0.3784 | 0.9294 | 3.71E-10 | 0.0188 | 273.2335 |
| rs2431697 | C | T | NCF2 | 0.0293 | -0.2231 | 0.9404 | 2.60E-14 | 0.0056 | 80.0685 |
| rs6889239 | C | T | TNIP1 | 0.0317 | 0.2776 | 0.2575 | 2.19E-18 | 0.0295 | 433.2189 |
| rs389884 | G | A | STK19 | 0.0432 | 0.9282 | 0.0736 | 2.92E-102 | 0.1175 | 1899.1543 |
| rs9274357 | T | C | HLA-DQB1 | 0.0352 | 0.4574 | 0.7763 | 1.28E-38 | 0.0727 | 1117.9017 |
| rs7768653 | T | C | ATG5 | 0.0297 | -0.207 | 0.4016 | 3.11E-12 | 0.0206 | 300.0028 |
| rs12524498 | T | G | HCP5 | 0.1208 | -0.6733 | 0.9891 | 2.48E-08 | 0.0098 | 140.8349 |
| rs58721818 | T | C | AL356739.1 | 0.0756 | 0.6575 | 0.9751 | 3.38E-18 | 0.021 | 305.9024 |
| rs150180633 | T | C | TBC1D22B | 0.069 | 0.9282 | 0.9831 | 2.66E-41 | 0.0286 | 420.4382 |
| rs28361029 | A | G | GRCh37 | 0.0614 | -0.3857 | 0.0101 | 3.27E-10 | 0.003 | 42.5522 |
| rs35000415 | T | C | IRF5 | 0.0415 | 0.5878 | 0.8996 | 1.86E-45 | 0.0624 | 949.5369 |
| rs2736332 | C | G | FAM167A | 0.0321 | 0.2776 | 0.2694 | 4.83E-18 | 0.0303 | 446.3747 |
| rs7823055 | T | G | RP1 | 0.0286 | -0.3507 | 0.4235 | 1.64E-34 | 0.06 | 911.1935 |
| rs7899626 | T | C | ARID5B | 0.0333 | 0.1823 | 0.6372 | 4.19E-08 | 0.0154 | 222.6638 |
| rs7097397 | A | G | WDFY4 | 0.0287 | -0.1863 | 0.3956 | 8.60E-11 | 0.0166 | 240.8347 |
| rs58688157 | G | A | CDHR5 | 0.0336 | -0.2231 | 0.7316 | 2.97E-11 | 0.0196 | 284.515 |
| rs353608 | G | A | AL356215.1 | 0.028 | 0.1863 | 0.5477 | 2.93E-11 | 0.0172 | 249.6733 |
| rs73050535 | T | C | RP11-429A20.3 | 0.1241 | -0.7134 | 0.9702 | 9.11E-09 | 0.0294 | 432.4694 |
| rs597808 | G | A | ATXN2 | 0.0295 | -0.1625 | 0.5338 | 3.51E-08 | 0.0131 | 190.0238 |
| rs1143679 | A | G | ITGAM | 0.04 | 0.5822 | 0.1312 | 5.03E-48 | 0.0773 | 1194.6825 |
| rs28834423 | C | G | ITGAM | 0.0365 | 0.4574 | 0.1829 | 5.65E-36 | 0.0625 | 951.6512 |
| rs13332649 | G | A | LINC02132 | 0.0376 | -0.3147 | 0.8022 | 5.43E-17 | 0.0314 | 462.9181 |
| rs143123127 | A | G | IKZF3 | 0.084 | 0.47 | 0.0308 | 2.23E-08 | 0.0132 | 190.6491 |
| rs35251378 | A | G | TYK2 | 0.0324 | -0.2357 | 0.2694 | 3.61E-13 | 0.0219 | 318.9951 |
| rs73068668 | A | G | PPP6R1 | 0.0575 | -0.3147 | 0.0954 | 4.40E-08 | 0.0171 | 248.0955 |
| rs3747093 | A | G | CCDC116 | 0.0345 | 0.2624 | 0.2018 | 2.88E-14 | 0.0222 | 323.5059 |

EA, effect allele; EAF, effect allele frequency; OA, other allele; SE, standard error; SNP, single nucleotide polymorphism; beta value equals log (OR).


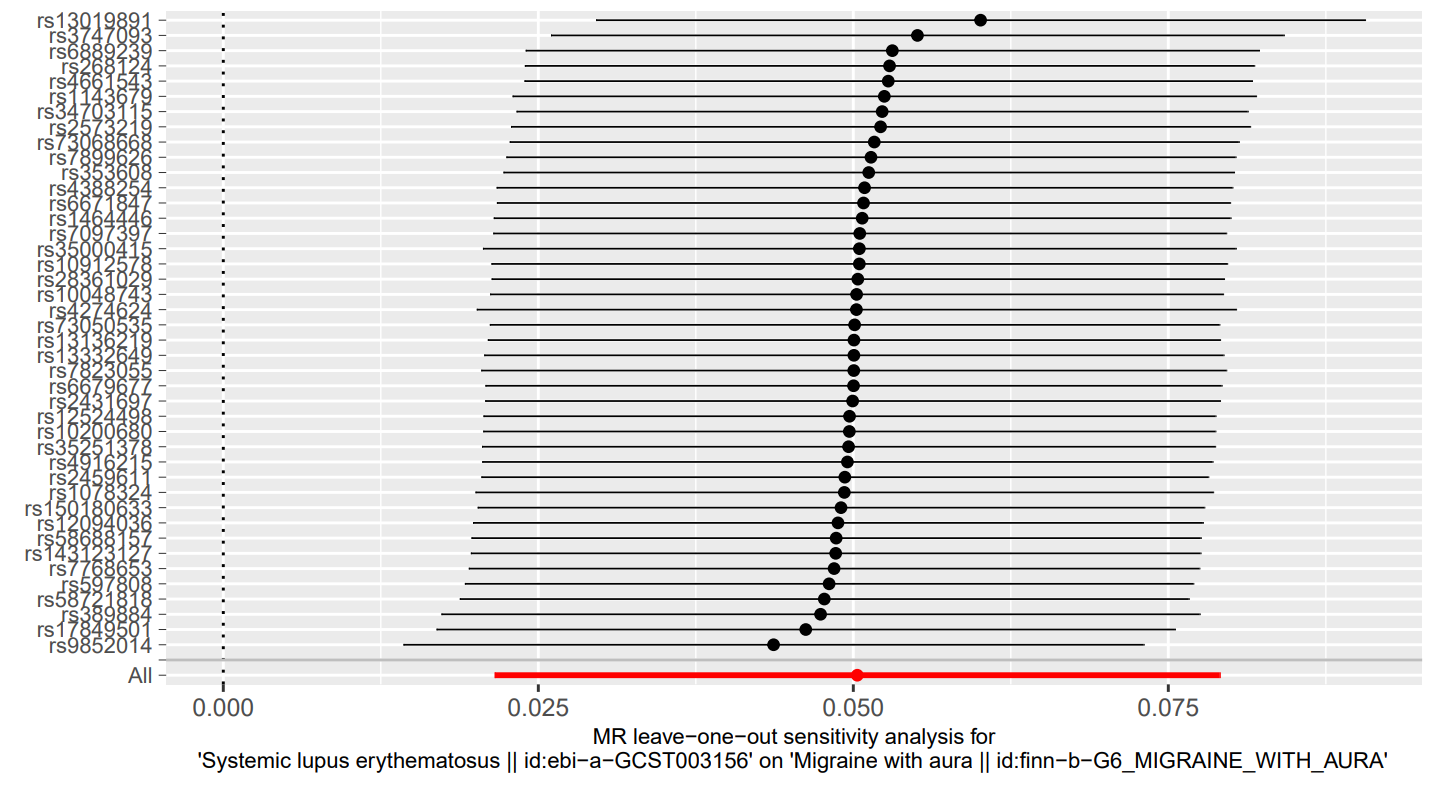


1. MR leave-one-out sensitivity analysis for SLE on the risk of migraine with aura. MR, Mendelian randomization; SLE, systemic lupus erythematosus.


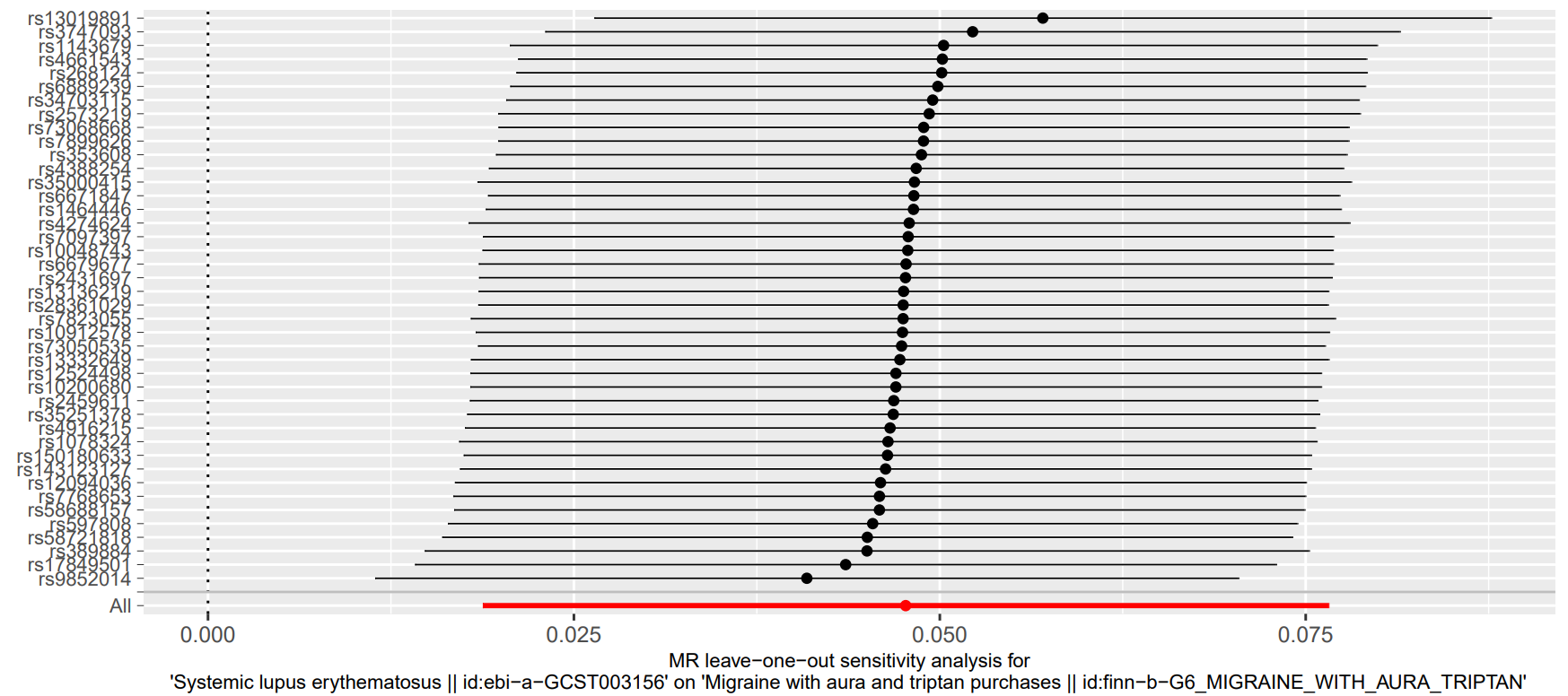


1. MR leave-one-out sensitivity analysis for SLE on the risk of migraine with aura and triptan purchases. MR, Mendelian randomization; SLE, systemic lupus erythematosus.


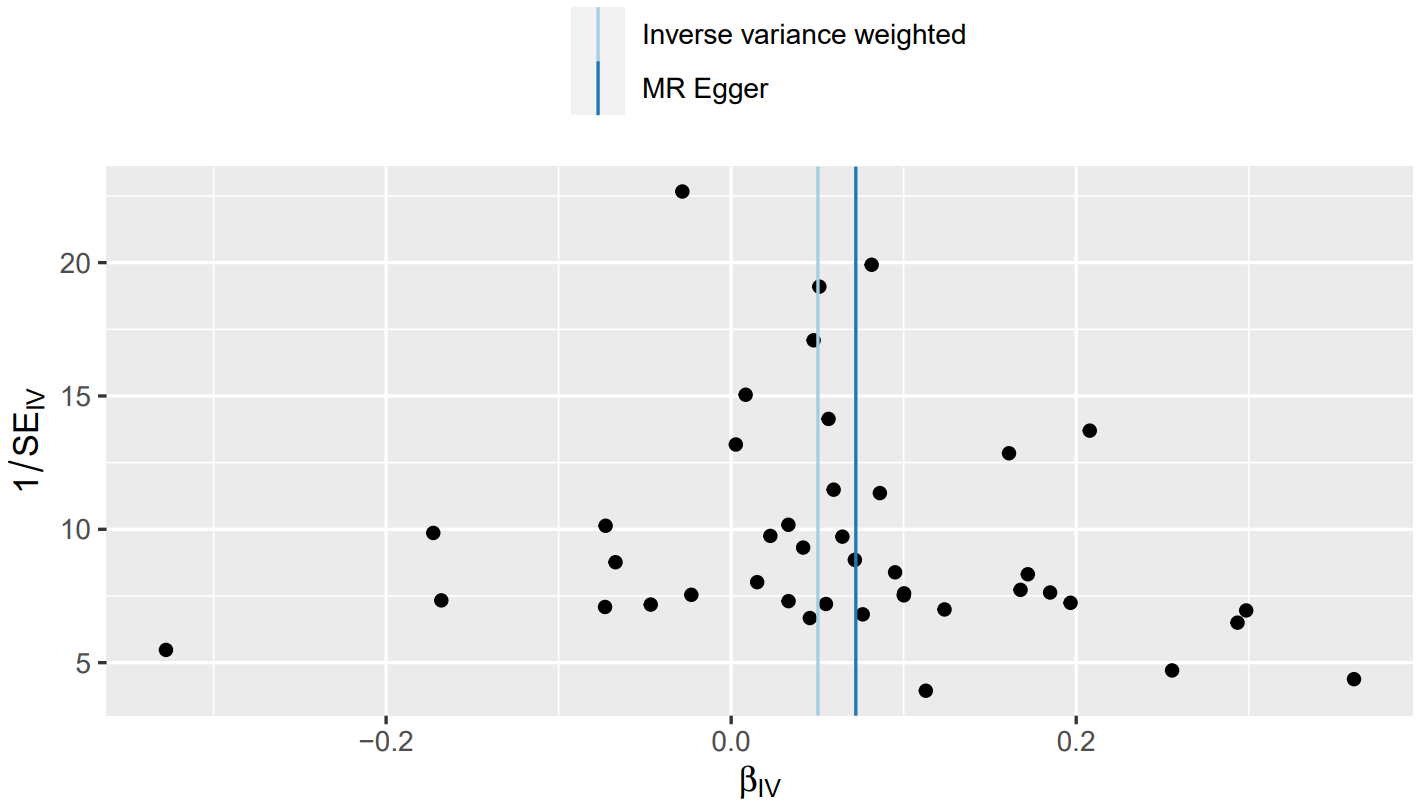


1. Funnel plot of causal effect of SLE on the risk of migraine with aura. IV, instrumental variable; MR, Mendelian randomization; SLE, systemic lupus erythematosus.


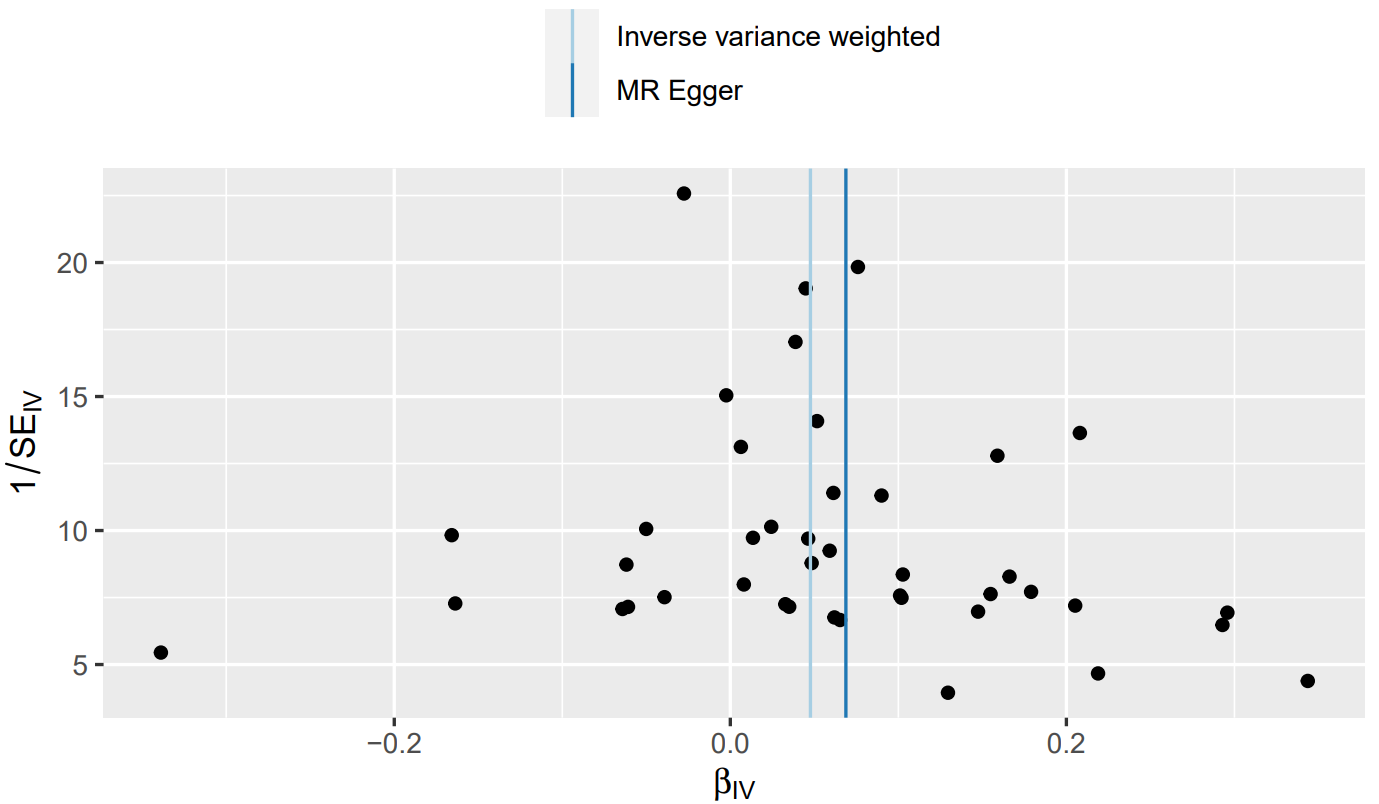


1. Funnel plot of causal effect of SLE on the risk of migraine with aura and triptan purchases. IV, instrumental variable; MR, Mendelian randomization; SLE, systemic lupus erythematosus.
